# Voltage-Gated Calcium Channel Antagonists and Traumatic Brain Injury

**DOI:** 10.3390/ph6070788

**Published:** 2013-06-26

**Authors:** Gene Gurkoff, Kiarash Shahlaie, Bruce Lyeth, Robert Berman

**Affiliations:** 1 Department of Neurological Surgery, One Shields Avenue, University of California, Davis, CA 95616, USA; E-Mails: kiarash_shahlaie@ucdmc.ucdavis.edu (K.S.); bglyeth@ucdavis.edu (B.L.); rfberman@ucdavis.edu (R.B.); 2 NSF Center for Biophotonics Science and Technology, Suite 2700 Stockton Blvd, Suite 1400, Sacramento, CA, 95817, USA

**Keywords:** voltage-gated calcium channels, antagonists, ziconotide, nimodipine, traumatic brain injury

## Abstract

Traumatic brain injury (TBI) is a leading cause of death and disability in the United States. Despite more than 30 years of research, no pharmacological agents have been identified that improve neurological function following TBI. However, several lines of research described in this review provide support for further development of voltage gated calcium channel (VGCC) antagonists as potential therapeutic agents. Following TBI, neurons and astrocytes experience a rapid and sometimes enduring increase in intracellular calcium ([Ca^2+^]_i_). These fluxes in [Ca^2+^]_i_ drive not only apoptotic and necrotic cell death, but also can lead to long-term cell dysfunction in surviving cells. In a limited number of *in vitro* experiments, both L-type and N-type VGCC antagonists successfully reduced calcium loads as well as neuronal and astrocytic cell death following mechanical injury. In rodent models of TBI, administration of VGCC antagonists reduced cell death and improved cognitive function. It is clear that there is a critical need to find effective therapeutics and rational drug delivery strategies for the management and treatment of TBI, and we believe that further investigation of VGCC antagonists should be pursued before ruling out the possibility of successful translation to the clinic.

## 1. Introduction

### 1.1. Significance of Traumatic Brain Injury

It is estimated that well over 5.3 million people live in the United States with deficits related to traumatic brain injury (TBI) [1], with over 1.7 new TBI cases annually. A recent meta-analysis examining the prevalence of TBI in the general adult population found that that approximately 12% of the general adult population has a history of TBI with loss of consciousness (16.7% for males and 8.5% for females) [2]. Many patients suffering severe [3,4,5] as well as mild or moderate TBI [5,6,7] are unable to return to work, maintain steady employment, and they struggle with daily tasks due to persistent cognitive deficits. In fact, in a recent study by the TBI Model Systems National Data Centre only 38% of patients followed were employed 2-years following their TBI [8]. Approximately $48.3 billion dollars is spent on TBI patient care in the US each year, with over $31.7 billion (65%) for those that survive [9]. The annual cost for new cases of acute TBI care and rehabilitation is estimated to be approximately $10 billion in the U.S. alone. To date, no effective pharmacotherapy has been shown to improve outcome following TBI, in spite of intensive research in this area [10]. Therefore, the need to develop effective drugs for TBI continues to be pressing. In this paper we review the role of voltage gated calcium channels (VGCC) in the pathophysiology of TBI, and evidence that antagonists of VGCC can be neuroprotective in animal models of TBI and may have potential for clinical use.

### 1.2. Regulation of Intracellular Calcium

Calcium ions (Ca^2+^) are major regulators of vital cellular functions. Through their interactions with specific calcium binding proteins, including calmodulin, parvalbumin, calbindin and calretinin, calcium ions are involved in the regulation of secretory functions (e.g., neurotransmitters, hormones), enzyme activity, intracellular transport, contractile processes, glycolysis, respiration, mitosis, membrane potential and intracellular communication [11,12,13,14,15]. These functions are critical for cell survival and disruption of calcium regulation can be catastrophic for the cell. It is therefore not surprising that interference with Ca^2+^ homeostasis contributes to cell injury and death in a number of pathological conditions, including traumatic brain injury [16]. In fact, calcium has been called the “final common pathway” for toxic cell death [17].

Intracellular cytosolic calcium levels are maintained at low resting levels between 50–100 nM via the combined activity of VGCC’s, receptor operated channels (ROC), store operated channels (SOC), calcium-ATPase transporters in the plasma membrane (PMCA) and smooth endoplasmic reticulum (SERCA), as well as the Na^+^/Ca^2+^ exchanger in endoplasmic reticulum, mitochondria and plasma membrane. Some inorganic calcium is also bound to bicarbonate, phosphate and phosphatides. Intracellular stores contained within the endoplasmic reticulum and mitochondria also influence cytosolic calcium levels. For example, receptor-generated inositol triphosphate (IP_3_) can release calcium from stores within the endoplasmic reticulum [18,19] and ryanodine receptor activation on the endoplasmic reticulum and mitochondria represent an additional important source of intracellular Ca^2+^ [20].

## 2. Voltage Gated Calcium Channels

### 2.1. Voltage Gated Calcium Channel Structure

**Figure 1:**
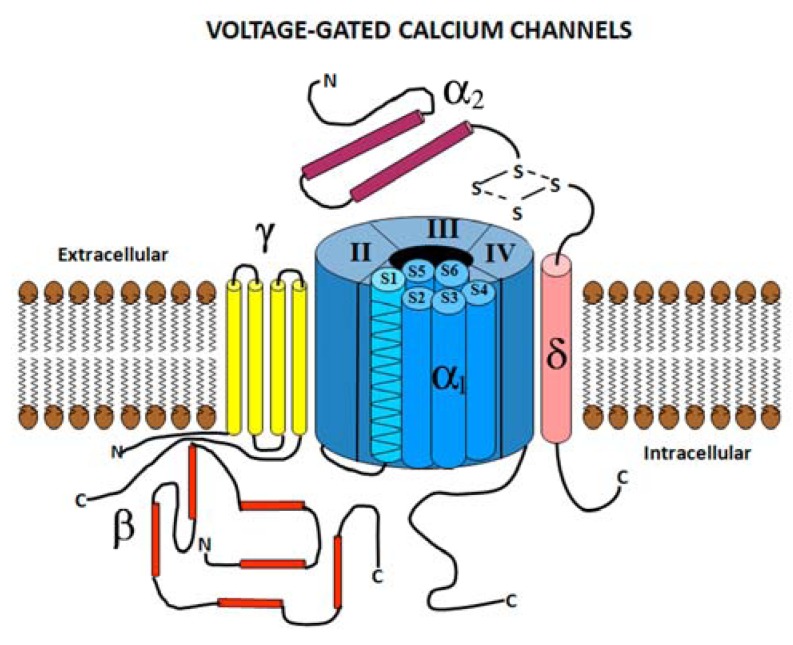
Subunit structure of voltage gated calcium channels (VGCC). The α1 is the pore-forming subunit which contains voltage-sensing machinery and the binding sites of channel blockers. α_1_ subunit contains 4 homologous domains (I–IV), each containing 6 transmembrane helices (S1–S6). The α2δ and β subunits enhance expression and modulate the voltage dependence and gating kinetics of VGCCs.

Voltage-gated calcium channels are heteromultimers formed by an α_1_ subunit and three auxiliary subunits α_2_-δ, β, and γ [21] (Figure 1). The α_1_ subunit is the largest (190–250 kDa) and incorporates the conduction pore, voltage sensor and gating apparatus. It is also the major site of channel regulation by second messengers, drugs, and toxins. The α_1_ subunit is comprised of four homologous domains composed of six transmembrane helical segments (S1–S6) that determine key channel characteristics [21]. For example, the S4 segment functions as the voltage sensor and the pore loop between segments S5 and S6 determines ion selectivity and conductance. Ten distinct α_1_ subunits have been described, associated with six different classes of VGCCs [22,23,24,25,26]. In addition there are also several ancillary proteins (β, α2δ, and γ) associated with the α1 subunit that form a multimeric complex (see Figure 1). These ancillary subunits modify the biophysical properties of the VGCC, second messenger modulation and intracellular transport [27,28]. Finally, there is a growing literature related to the structure and function of splice variants of the L- [29,30], N- [31], P/Q- [31], R- [32] and T-type [33] VGCCs. In fact there is evidence that alternate splice variants of both the L- [34,35] and N-type [36] VGCC can influence the affinity and/or sensitivity of channels to specific antagonists.

### 2.2. Voltage Gated Calcium Channel Subtypes

Classification of the six subtypes of VGCCs was originally based on their biophysical and pharmacological properties (Table 1). They are typically referred to as L-, N-, P-, Q-, R- and T-type channels [22,23,25,37]. However, a newer gene-based nomenclature is in use [38]. Under the new nomenclature L-type channels are designed Ca_v_1.1-1.4, P/Q as Ca_v_ 2.1, N as Ca_v_ 2.2, R as Ca_v_ 2.3, and T-type channels as Ca_v_3.1-3.3. It addition there is evidence of addition R-type Ca^2+^ current that is separate from the Ca_v_ 2.3 channel [39]. 

**Table 1:** Summary of the properties and common antagonists of voltage-gated calcium channel blockers.

Channel Type	L Ca_v_1.1-1.4	N Ca_v_2.2	P Ca_v_2.1	Q Ca_v_2.1	R Ca_v_2.3	T Ca_v_3.1-3.3	Ref.
Conductance (pS)	25	11 to 20	9 to 20	15 to 16	15 to 20	8	[40]
Selectivity (Ca^2+^>Ba^2+^)	2:1	2:1	2:1	ND	1.3:1	1:1	[40]
Activation Potential (mV)	−10 to −50	−20	−50	−50	−25 to −40	−70	[40]
Inactivation Kinetics (msec)	150–2,000	100–200	500–1,000	500–1,000	50–100	10–70	[40]
**Calcium Blockers (IC50)**							
ω-conotoxin MVIIA	None	78 nM–1 µM	None	None	None	None	[40,41,42]
ω-conotoxin GVIA	None	28 nM–2 µM	None	None	None	None	[40,41,42]
ω-Agatoxin AgaIVA	None	None	15 nM	50 nM–1 µM	50 nm	None	[41,43]
ω-conotoxin MVIIC	None	18 nM	18 nM	50 nM–1 µM	None	None	[41,44]
ω-Agatoxin AgaIIIA	1 nm	1 nm	IC50 N/A	IC50 N/A	None	None	[45]
SNX-482	None	None	30–750 nm	30–750 nm	15–30 nM	None	[41,46,47]
Nimodipine	0.135–2.6 µM	None	None	None	None	5–11 µM	[48,49,50,51]
Nifedipine	100 nM	None	None	None	None	39 µM	[50]
Efonidipine	10 µM	None	None	None	None	1.3–13 µM	[51,52,53]
Amplodipine	3–5 µM	None	None	None	None	4–13 µM	[51,54,55]
Nicardipine	9–26 µM	None	32–97 µM	32–97 µM	None	5–13 µM	[55]
Verapamil	0.6–1 µM	None	None	None	None	20–30 µM	[49,56,57]
Diltiazem	3–33 µM	None	None	None	None	30 µM	[49,57]
Mibefradil	1.7–21 µM	None	208 µM	208 µM	None	0.5–11 µM	[51,58,59]

For simplicity we will retain the older nomenclature in this review (*i.e.*, L, N, P, Q, R & T). As shown in the table above, the various channels differ in their conductance rates, voltage activation threshold, and rate of inactivation. A complete review of calcium channel biophysics is beyond the scope of this review. However, L-type channels are high-threshold, large-conductance, slowly inactivating calcium channels [35]. The L-type channel is well understood pharmacologically and defined by its sensitivity to blockade by dihydropyridines such as nimodipine. The N-, P, Q and R-type voltage gated calcium channels are intermediate, having medium conductances, intermediate inactivation kinetics, and medium to high activation thresholds [21]. These calcium channels are selectively blocked by various snail and spider toxins as summarized above. The T-type channel is a low-threshold, low-conductance, rapidly inactivating calcium channel [60]. T-type channels can be blocked by Mibefradil with a 10–30 times higher potency than L-type channels [61,62]. All six types of VGCC’s are found in brain, with the L, N-, P- and Q- and R-type channels involved in synaptic transmission [21]. The N, P, Q and R-type channels are also critically involved in regulating calcium-dependent neurotransmitter release from presynaptic terminals, while the L- and T-type channels appear to make little contribution to this process [24]. The low-voltage activated T-type channel appears to be primarily involved in the generation of rhythmic burst firing of neurons, and is thought to contribute to network synchrony and epilepsy [63].

### 2.3. Voltage Gated Calcium Channel Distribution in the Nervous System

The distributions of VGCCs in mammalian brain have been examined by receptor autoradiography [64,65] and immunocytochemistry [66,67,68]. L-type VGCC’s are widely distributed in muscle, endocrine cells and brain, but are less abundant in neuronal tissues compared to other VGCC’s [23]. N-type VGCC’s show a wide distribution in rodent brain, with highest densities found in cerebral cortex, dendritic zones of the hippocampus, amygdala, septal nuclei, medial geniculate, superior colliculus, molecular layers of cerebellar cortex, n. solitary tract and spinal cord (layers 1–3) [23,64,66,67,69]. P/Q-type calcium channels, although not as widely distributed as N-type channels, are located on cerebellar granule cells, interneurons and Purkinje cells, and in the hippocampus on pyramidal and granule cells [25,66]. While less well described, there is evidence of R-type VGCC in the cortex [70], thalamus [71], and hippocampus [72,73]. T-type VGCC’s have been demonstrated in cerebellum, thalamus, olfactory bulb and hippocampus [74]. At the cellular level N and P/Q-type VGCC’s are found both pre- and post-synaptically, and interact directly with presynaptic core proteins syntaxin and SNAP-25, providing a molecular basis for Ca^+2^ influx into nerve terminals and transmitter release [75,76]. Astrocytes have L- and possibly T-type channels, while, to date, VGCCs have not been observed on mature oligodendrocytes [77].

### 2.4. Characterizing Voltage Gated Calcium Channels Based on Pharmacology

Dihydropyridine antagonists (e.g., nimodipine, nifedipine, verapamil, amlodipine) robustly block L-type channels [78]. While originally it was thought that the remaining VGCC were dihydropyridine insensitive, it was demonstrated in *Xenopus* oocytes that several of these blockers (including amlodipine) also antagonized N-, P- and Q-type channels at higher concentrations [55]. It was also observed that the dihydropyridine efonidipine inhibits not only the L- but also the T-type VGCC [79]. Finally, it has recently been suggested that, at the concentrations used, the effect of dihydropyridines on the R-type channel have been occluded. Specifically, at a 500 nm concentration, isradipine was able to partially antagonize the R-type current in cardiomyocytes [80]. In conclusion, while the ability of dihydropyridines to antagonize L-type channels is undisputed, these compounds appear to have effects across the spectrum of VGCC. It is clear, therefore, that more research is warranted into the antagonism of each of the VGCC subtypes by dihydropyridines. The N-, P- and Q-type channels can be blocked by specific ω-conotoxins and ω-agatoxins [24,25,81]. The ω-conotoxins or ω-conopeptides are basic, water soluble, 24–29 amino acid peptides isolated from the venom of fish-hunting marine snails belonging to the genus *Conus* [81]. The best-characterized are the ω-conotoxins GVIA, TVIA (SNX185) and MVIIA (*i.e.*, SNX111) that block the N-type calcium channel [24,82]. A large number of highly selective analogs (e.g., SNX111 and SNX185) have been synthesized and carry the designation “SNX” in the literature. The ω-agatoxin AgaIVA is isolated from the venom of funnel-web spiders and is a potent blocker of P- (K_d_1-3 nM) and Q-type (K_d_90 nM) VGCC’s. The P- and Q-type VGCC’s are differentiated pharmacologically by their sensitivity, but not selectivity to AgaIVA [24]. The N-, P- and Q-type channels are all blocked by the ω-conotoxin MVIIC [24]. The R-type VGCC’s are resistant to ω-conotoxins and ω-agatoxins, but have been reported to be blocked by a selective inhibitor SNX-482 although the selectivity of SNX-482 for T-type VGCCs has recently been challenged [83,84,85]. T-type blockers include several dihydropyridine antagonists including efonidipine, felodipine and nitrendipine [51,61,86] as well as nimodipine [51]. Mibefradil has also been identified as a potent T-type channel blocker [87]. There is also evidence that specific heavy metals have the ability to block VGCC including lead for L-type [88], nickel [89] and zinc [90] for T-type, and zinc and copper for R-type channels [91]. To date, antagonism of either L-Type (α_1S_ and α_1D_) or N-type (α_1B_) channels has been evaluated as a strategy for improving outcome following TBI, but similar studies have not yet been carried out with blockers of P/Q, T or R-type VGCCs.

## 3. Experimental Evidence that Blockade of VGCC’s Can be Neuroprotective

### 3.1. Pathological Calcium Accumulation Following TBI

The pathophysiology of TBI has typically been separated into primary and secondary injuries. Primary injury occurs at the time of impact as a result of mechanical tissue deformation resulting in contusions, lacerations, shearing of axonal connections and hemorrhage. Primary injury also initiates a cascade of secondary injury mechanisms, including a large influx of calcium into damaged cells, which can trigger further cell death and lead to substantially increased morbidity [17,92,93,94,95,96]. 

**Figure 2:**
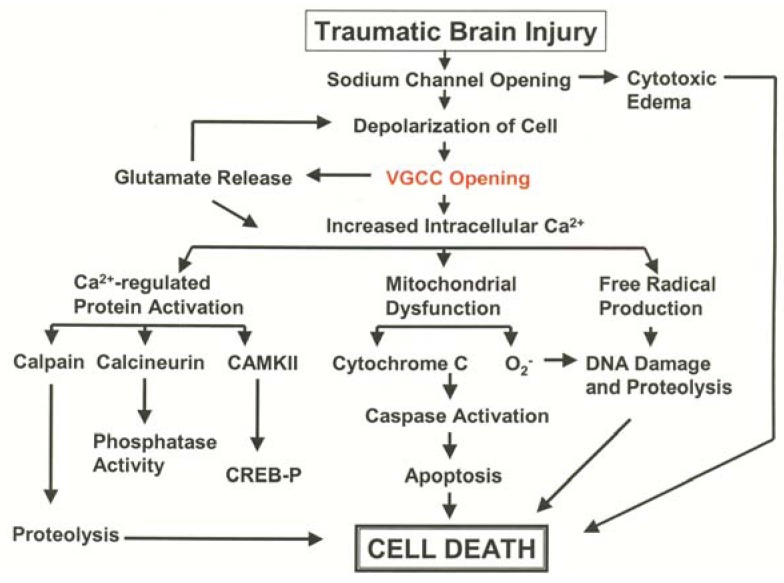
Schematic describing the role of VGCC in TBI-induced neuronal and astorcytic cell death.

There is ample experimental evidence that intracellular calcium overload occurs after brain injury, and is a key early step in the activation of secondary injury mechanisms (Figure 2) [17,92,93,94,95,96]. Several *in vitro* studies have documented a large increase in [Ca^2+^]_i_ that occurs following traumatic injury to neurons and astrocytes in culture [97,98,99,100,101,102,103,104,105,106,107]. Calcium influx from injury in neurons can result from activation of the N-methyl-D-Aspartate (NMDA) receptor [105,108,109,110,111], opening of VGCCs [102,110,112], as well as release from intracellular stores [16,103,113]. Accumulation of [Ca^2+^]_i_ in astrocytes has also been demonstrated to come from release from intracellular stores [99,113] as well as the activation of the sodium calcium exchanger [98]. Many *in vitro* studies have now demonstrated that disruption of calcium homeostasis can injure cells or lead to cell death and have implicated a variety of cellular mechanisms (Figure 2). These include in activation of apoptotic pathways [114,115,116,117,118,119], mitochondrial dysfunction [120,121,122,123,124], free radical production [125] lipid peroxidation [126,127] and osmotic disturbances [128]. In cultures, mechanical strain injury not only increased acute [Ca^2+^]_i_, but also triggered delayed depolarization lasting up to 24 h following injury [129]. Consequences of increased calcium uptake include significant neuronal [100,105,112,130,131] and astrocytic [98,101,132] cell death as well as persistent neuronal dysfunction [133,134,135,136,137,138]. Second insults such as hypoxia [112,139], and ischemia [139,140,141,142,143] can lead to additional and extended accumulation of [Ca^2+^]_i_ which is associated with further diminished outcome [112].

*In vivo* studies, although far fewer in number, also support the involvement of calcium influx following traumatic injury to the spinal cord and brain. Extracellular calcium levels fall dramatically immediately after spinal cord injury [95,144], while total tissue levels of calcium increase [107]. Nilsson and colleagues, using calcium sensitive electrodes, identified immediate and dramatic decreases in extracellular calcium at the focus of injury in a weight-drop model of cortical contusion in rats. The authors concluded that massive Ca^2+^ entry into the intracellular compartment occurring at the site of injury was responsible for this phenomenon [145]. Dienel [146] used ^45^Ca^2+^ autoradiography to map increased cellular levels of calcium after transient forebrain ischemia in rats, and argued that such results were due to an accumulation of intracellular calcium after injury. Following lateral fluid percussion, assessment of ^45^Ca^2+^ using autoradiography indicated not only an acute increase of calcium in the injured cortex and hippocampus, but a sustained accumulation lasting for several days post-injury. Furthermore, a more diffuse ^45^Ca^2+^ accumulation was also detected in the thalamus that could be observed for up to two weeks following injury [147,148]. Using the lateral fluid percussion model of TBI, DeLorenzo and colleagues acutely isolated injured hippocampal neurons in culture and imaged calcium fluxes using the calcium sensitive dye Fura-2-AM. In these preparations [Ca^2+^]_i_ levels remained elevated for at least one week following fluid percussion TBI and calcium homeostasis was impaired for at least 30 days, indicating that TBI has enduring effects on Ca^2+^ dynamics [149,150]. These pre-clinical studies document the significant increase in [Ca^2+^]_i_ that follows TBI, and point to the possibility that pharmacological interventions aimed at reducing the rise in [Ca^2+^]_I_, such as antagonism of VGCC (Figure 2), could be a rational and effective therapeutic strategy to reduce cell death and dysfunction.

### 3.2. L-Type VGCC Antagonists to Treat Traumatic Brain Injury

The L-type VGCCs require strong depolarization for activation, and are typically related to long-lasting calcium currents [21]. L-type VGCCs are prominent in the vertebrate cardiac, skeletal and smooth muscle. In the central nervous system, they are located on the cell body and proximal and distal dendrites [66,151]. There are three classes of L-type VGCC antagonists including phenalkylamines (verapamil, D-600), benzothiazepine (dlitiazem) and dihydropyridines (nimodipine, nifedipine, nitrendipine) [21]. Pre-clinical rodent [152,153,154,155] and primate [156] studies demonstrated the utility of nimodipine to improve cerebral blood flow, reduce infarct volume and improve behavioral outcome following an ischemic injury. Importantly, improved outcome was observed in the absence of significant drug side effects. Clinical trials to translate nimodipine to treat the acute effects of ischemia found significant improvements in both blood flow and outcome following injury [157,158,159]. 

Despite the promising clinical evidence for the potential of L-type antagonists to improve outcome following an ischemic insult, there is significantly less data evaluating these compounds in traumatic brain injury. Initial *in vitro* studies found that nimodipine reduced cell death following treatment with high concentrations of glutamate [160]. In *in vitro* cell culture models of uniaxial and bi-axial mechanical strain injury, a cocktail of a sodium channel blocker (tetrodotoxin, TTX) NMDA antagonist ((2*R*)-amino-5-phosphonopentanoate; APV), AMPA antagonist (6-cyano-7-nitroquinoxaline-2,3-dione; CNQX), and nimodipine applied prior to strain significantly reduced the average accumulation of [Ca^2+^]_i_ in neurons as well as the total number of neurons experiencing significant [Ca^2+^]_i_ load [130]. In a different model of mechanical injury, nifedipine significantly reduced cell death and improved cell function in an organotypic hippocampal slice culture [97]. In initial *in vivo* studies, verapamil administered following a lateral fluid percussion traumatic brain injury in rats significantly improved cerebral blood flow in the injured cortex leading the authors to conclude that L-type VGCC play a role in vasoconstriction and the loss of vasoreactivity following TBI [161]. In a separate study, nimodipine treatment was able to reduce lipid peroxidation caused by traumatic brain injury in rodents [162]. Considering the extensive literature on the accumulation of [Ca^2+^]_i_ and the loss of Ca^2+^ homeostasis, there is sparse pre-clinical data evaluating the potential of L-type VGCC antagonists to improve outcome following TBI.

Even with such limited pre-clinical evidence, L-type VGCC antagonists have been tested in several clinical trials in patients with severe TBI examining their potential to improve outcome when administered systemically acutely following injury. In initial clinical trials (HIT 1) it was observed that intravenous delivery of nimodipine had modest effects on outcome with only a trend toward improved outcome [163,164]. The authors also noted that the drug was well tolerated and patients experienced few side effects. In a follow up study (HIT II) [165] these same authors reached a similar conclusion that nimodipine had modest effects on patients with severe TBI. However the authors made an additional observation; nimodipine was more efficacious in patients with evidence of traumatic subarachnoid hemorrhage from their initial CT scan. However subsequent studies have failed to find a similar beneficial effect even within this subpopulation [166,167]. A more recent study reported that cerebral metabolism and outcome were improved in a small patient population by intravenous nimodipine treatment [168,169]. Therefore, there continues to be considerable uncertainty about a beneficial effect of nimodipine treatment for acute traumatic brain injury [170]. In summary, to date, antagonists of L-type VGCC have failed to provide convincing evidence that blockade of L-type VGCCs is an effective therapeutic strategy for TBI.

### 3.3. N-Type VGCC Antagonists to Treat Traumatic Brain Injury

N-type VGCCs are activated by strong depolarization, and are found on the dendrites, soma, and axon terminals of neurons [67,171,172,173]. These VGCCs located at axon terminals play a critical role in neurotransmitter release throughout the central nervous system [172,174], and may play a dominant role in norepinephrine release from sympathetic neurons [175]. In 1985 Olivera and colleagues described small peptides isolated from the venoms of cone snails that were very specific and potent inhibitors of N-type VGCCs [25,69,81]. One sub-class, the ω-conotoxins, MVIIA was found to be highly selective for the N-type VGCC, and a synthetic form of the toxin called ziconotide (also known as SNX-111, Prialt^®^) was developed and has been used in studies of pain systems, ischemia and TBI [176,177,178,179]. Similar to L-type antagonists, initial characterization of ziconotide was on improving outcome in rodent models of ischemia [180,181,182]. Unlike L-type antagonists, however, the focus of ziconotide treatment was neuroprotection and not blood flow.

Because of their role in neurotransmitter release, N-type VGCCs represent an attractive target for neuroprotection through their ability to reduce excitatory neurotransmitter release and there by prevent or limit excitotoxic cell damage by glutamate. However, there have been relatively few pre-clinical studies of the potential of N-type VGCC antagonists in experimental models of TBI. A recent *in vitro* study, using a mechanical strain-injury model of TBI in mixed neuronal glial cultures, examined the effects of the selective N-type VGCC blocker SNX-185 on [Ca^2+^]_i_ imaged with Fura-2-AM and neuronal and astrocyte survival after injury by immunohistochemistry. SNX-185 is a synthetic form of ω-conotoxin TVIA and is similar to MVIIA (*i.e.*, ziconotide) in its selectivity for N-type VGCCs. Addition of SNX-185 to the culture media before or immediately (*i.e.*, <5 sec) after mechanical strain-injury significantly reduced the rise in [Ca^2+^]_i_ and improved survival of injured neurons and astrocytes following mild, moderate or severe mechanical strain injury [102]. Concentrations of 100 and 1000 nM SNX-185 in the culture media appeared to be equally effective. Delayed addition of SNX-185 to the media 5 min after mechanical strain injury did not improve cell survival nor did it prevent the rise in [Ca^2+^]_i_. However, these results would be expected because in the *in vitro* mechanical strain-injury model the peak increase in [Ca^2+^]_i_ occurs within a few seconds to a minute after injury, before the 5 min delayed treatment with SNX-185. However, mechanical injury does lead to extended elevations in [Ca^2+^]_i_ [16,104,105]. Interestingly, treatment with SNX-185 facilitated the return of injured neurons to more normal calcium homeostasis. Specifically, elevated levels of [Ca^2+^]_i_ following strain injury returned to near baseline levels (*i.e.*, 100 nM) significantly sooner in injured neurons when SNX-185 was added to the bath media 5 min after injury. 

Because [Ca^2+^]_i_ can remain elevated for long periods of time after injury, these results suggest that that N-type VGCC blockers may prevent the rise in [Ca^2+^]_i_ for cells that may be injured but show a delayed rise in [Ca^2+^]_i_ and facilitate the return of calcium homeostasis. However, it remains to be demonstrated experimentally whether calcium homeostasis is normal or whether or not the return to baseline calcium levels would be beneficial to injured cells. Microdialysis samples taken from these cultures demonstrated a significant reduction in extracellular glutamate [102] suggesting that, in part, SNX-185 was decreasing calcium accumulation by blocking presynaptic N-type calcium channels and thereby reducing pre-synaptic glutamate release. Recently, these same authors tested whether SNX-185 would have similar effects in an *in vitro* model of mechanical injury with hypoxia as a second insult [112]. Hypoxia was produced by changing the cell culture CO_2_ level from 5% (normoxic) to 20% (*i.e.*, hypoxic) after mechanical strain injury, and then carrying out calcium imaging with Fura-2-AM 3, 6 and 24 h later. Compared to the normoxic condition, hypoxia further increased the rise of [Ca^2+^] after injury at all three imaging time points, and resulted in greater cell death across all mechanical strain injury severities. Addition of 1µM SNX-185 5 min after the second insult reduced the added effects of hypoxia on [Ca^2+^] as well as the effects of hypoxia on cell survival, resulting in great numbers of surviving neurons. These results point to the importance of secondary insults in the pathophysiology of TBI and further support continued research and development of N-type VGCC antagonists to improve outcome in TBI patients at risk for second insult. In an *in vitro* model of axonal injury in which the opening of stretch-activated sodium channels in axons was followed by an increase in calcium accumulation, damage to the injured axon was prevented by either blocking sodium channels with tetratrodotoxin or blockade of VGCCs by the omega-conotoxin MVIIC [106].

*In vivo* studies with ziconotide, the synthetic version of MVIIA, have also demonstrated neuroprotective activity in rodent models of TBI. When administered i.v. 1 h following lateral fluid percussion TBI, ziconotide significantly reduced the increase in ^45^Ca^2^ accumulation in the dorsal hippocampus for up to 48 h following injury, with less dramatic effects observed in the cortex [183]. In a follow up study the same authors found that intravenous ziconotide dramatically reduced ^45^Ca^2+^ accumulation in the ipsilateral cortex (by 75%), but also several other brain regions including ipsilateral thalamus and hippocampus (up to 50%) [184]. In the rat model of controlled cortical impact TBI, an accumulation of Ca^2+^ was found in mitochondria along with significant mitochondria respiratory dysfunction for up to 48 h following injury [124]. Intravenous administration of ziconotide within 4 h from the time of injury significantly improved mitochondrial respiration in the injured brain [185,186,187]. Mitochondrial function was restored even when ziconoitde was delivered 10 h following the lateral fluid percussion injury [185]. Repeated intravenous injections of ziconotide at 3, 6, and 12 h after an impact-acceleration model of diffuse axonal traumatic brain injury in rats significantly improved cognitive and sensory-motor function [179]. In a more recent study, direct stereotaxic injection of SNX-185 into the dorsal hippocampus 5 min following lateral fluid percussion brain injury significantly reduced neuronal loss in the ipsilateral hippocampus and improved motor and cognitive function [188]. However, in a recent study, extracellular glutamate was significantly elevated in the striatum for up to 48 h following midline fluid percussion TBI in rats [189], and the rise in glutamate was not blocked by the N-type VGCC blocker GVIA, suggesting that the elevated striatal glutamate following TBI may not be calcium-dependent. 

Although the preclinical data were limited and the exact neuroprotective mechanisms of ziconotide were not well understood, a phase II human safety, efficacy, and feasibility trial of intravenous ziconitide in severe TBI patients was initiated. Unfortunately the study was halted before completion due to complicating cardiovascular (*i.e.*, hypotension) effects of systemic ziconotide treatment, likely due to the effects of N-type antagonists on heart rate and blood pressure [190,191], and the results of this trial have not been published. Therefore, the potential for clinical development of N-type VGCC blockers remains an open question, one that should be further explored given the established role of calcium in cell injury following TBI, the established efficacy of N-type VGCC blockers in limiting intracellular calcium accumulation, and the strength of the preclinical data in *in vitro* and *in vivo* studies.

## 4. Conclusions

### 4.1. Lessons to be Learned from Ziconotide Development for Chronic Pain

To date there has been a failure to translate pre-clinical therapeutic strategies from bench to the bedside to treat patients with TBI. A great deal has been written about this dilemma and several recommendations have come from careful analyses of recent failures [10]. First, experimental treatments should target known mechanisms of secondary injury following TBI and the dosages used should be adequate to block the targeted injury mechanism. Second, the effectiveness of the treatment should be demonstrated in animal models of brain injury, and the therapeutic window and optimal duration of treatment should be established. Third, valid outcome measures should be used. Finally, safety of the treatment for eventual use in patients with TBI should be demonstrated. 

These recommendations and the development and clinical approval of intrathecal ziconotide for treatment of intractable pain offer lessons for the development of drugs, such as ziconotide, for use in TBI. As in TBI studies, initial findings in rodents [176,177,178,192] and in primates [176] indicated the potential for ziconotide to reduce chronic pain in the absence of significant side effects. However, initial trials to translate ziconotide to the bedside were not successful due to significant peripheral side effects of the calcium channel blocker in a limited clinical study, including substantial hypotension following systemic administration [193]. However, rather than abandoning zicontide for pain management, the intrathecal route of administration was adopted that provided pain relief at a lower dose and did not produce significant cardiovascular or central CNS effects. Currently intrathecal ziconotide, now available as Prialt, has been used successfully in patients to treat acute post-operative pain [194], AIDS-related pain, cancer-related pain [195], and severe chronic pain [196]. In the most recent trials, ziconotide was found to be both efficacious and safe for continuous administration over at least a three-year period [197,198]. Based on the evidence reviewed above it would seem reasonable to continue development of N-type VGCC blockers, including ziconotide and SNX-185, to improve motor and cognitive function following TBI.

### 4.2. Summary

Based on the limited pre-clinical data, we feel that specific L- and N-type VGCC antagonists continue to have translational potential for pharmacotherapy in TBI patients. Furthermore, there have been no specific studies addressing the role that P/Q, R and T-type VGCC play in the pathophysiology of traumatic brain injury. The mechanisms of action for antagonists for each of these channels have been well defined, and their bioavailability and pharmacokinetics have been characterized. There is substantial preclinical data supporting further development of N-type VGCC blockers for use in TBI [199]. To date, however, only a very limited number of pre-clinical experiments have evaluated the potential of either L- or N-type VGCC antagonists to improve outcome following TBI. There is also evidence that R-type VGCC may increase the constriction of arteries improving outcome in rodent models of subarachnoid hemorrhage [200] and that the R-type antagonist SNX-482 can improve blood flow in the days following injury [201]. Future studies should also include other N-type VGCC blockers such as SNX-185, antagonists of other VGCC (*i.e.*, P/Q, R or T-type channels), the use of lower doses to avoid or reduce complications due to systemic side effects. In addition, as demonstrated in the treatment for chronic pain, intrathecal delivery of ziconotide can reduce the effect of channel antagonists on peripheral targets such as the heart and vasculature. This is relevant not only for N-type channels but also R- [200,202], T- [202], P/Q- [202,203,204] and L-type channels [202,205] that also may alter global heart rate and/or blood flow when administered systemically. There may also be potential for VGCC antagonists to improve function following TBI in cases where patients endure second insults, such as hypoxia, ischemia or seizures. However, based on the recommendation from Bullock and colleagues [10], it is clear that further pre-clinical evaluation is necessary in order to design a well-informed clinical trial.
